# Automatic identification of incidents involving potential serious injuries and fatalities (PSIF)

**DOI:** 10.1038/s41598-024-58824-y

**Published:** 2024-04-06

**Authors:** Pulkit Parikh, Julia Penfield, Marc Juaire

**Affiliations:** VelocityEHS, 222 Merchandise Mart Plaza, Suite 1750, Chicago, IL 60654 USA

**Keywords:** Risk assessment, Risk identification, Potential serious injuries and fatalities, Natural language processing, Engineering, Computer science

## Abstract

Safety incidents have always been a crucial risk in work spaces, especially industrial sites. In the last few decades, significant efforts have been dedicated to incident control measures to reduce the rate of safety incidents. Despite all these efforts, the rate of decline in serious injuries and fatalities (SIFs) has been considerably lower than the rate of decline for non-critical incidents. This observation has led to a change of risk reduction paradigm for safety incidents. Under the new paradigm, more focus has been allocated to reducing the rate of critical/SIF incidents, as opposed to reducing the count of all incidents. One of the challenges in reducing the number of SIF incidents is the proper identification of the risk prior to materialization. One of the reasons for risk identification being a challenge is that companies usually only focus on incidents where SIF did occur reactively, and incidents that did not cause SIF but had the potential to do so go unnoticed. Identifying these potentially significant incidents, referred to as potential serious injuries and fatalities (PSIF), would enable companies to work on identifying critical risk and taking steps to prevent them preemptively. However, flagging PSIF incidents requires all incident reports to be analyzed individually by experts and hence significant investment, which is often not affordable, especially for small and medium sized companies. This study is aimed at addressing this problem through machine learning powered automation. We propose a novel approach based on binary classification for the identification of such incidents involving PSIF (potential serious injuries and fatalities). This is the first work towards automatic risk identification from incident reports. Our approach combines a pre-trained transformer model with XGBoost. We utilize advanced natural language processing techniques to encode an incident record comprising heterogeneous fields into a vector representation fed to XGBoost for classification. Moreover, given the scarcity of manually labeled incident records available for training, we leverage weak labeling to augment the label coverage of the training data. We utilize the F2 metric for hyperparameter tuning using Tree-structured Parzen Estimator to prioritize the detection of PSIF records over the avoidance of non-PSIF records being mis-classified as PSIF. The proposed methods outperform several baselines from other studies on a significantly large test dataset.

## Introduction

Effective risk management is not only vital to the safety of a workplace and the environment surrounding it but can also improve the competitive value and reputation of the organization^1^. A key aspect of risk management is risk assessment, which involves identifying potential hazards and assigning risk levels to them. The findings of risk assessment are weighed, along with other factors, by risk managers to support decision-making processes. While risk assessment and management was established as a scientific field relatively recently, it has attracted rapidly growing interest. This paper deals with data-driven risk identification. We note that the term risk only implies safety risk in the paper; we do not take into account other types of risk such as financial risk.

There exists a substantial amount of data regarding incidents at workplaces that caused injuries to workers, damage to property, and/or harm to the environment. Reports describing incidents provide valuable insights into the causes and consequences of incidents, enabling a more informed approach to risk identification. For a reasonably large workplace, the volume of the incident data makes it very time-consuming to manually sift through and analyze it to infer patterns and gain insights that can aid in risk identification. Moreover, it requires employees with special expertise, a scarce resource, to carry out such a manual analysis. In this paper, we focus on automatic risk identification from incident reports powered by machine learning.

Performing a risk-related predictive modeling task such as risk identification on incidents at workplaces can be challenging on two counts. Firstly, the data describing the incidents can be very heterogeneous. It is not inconceivable for incident data to involve textual data, images, categorical fields, numerical readings, and date and time information. Moreover, the textual fields can also vary, ranging from truly free-text fields containing multiple natural language sentences to fields involving only phrases with a small vocabulary size. It is also possible for one field to combine contents associated with multiple data types. A simple illustration of such a field is one measuring distance containing a numeric value and the associated unit, which could be one of several categorical values. The field can also contain multiple numeric values and units (e.g., “3 feet and 5 in.”). Another aspect that can make it challenging to carry out risk identification on incident data is the limited availability of relevant labeled data. Supervised learning is a commonly used form of machine learning for this task. It is hard to achieve good performance using supervised learning if the labeled data available for training is inadequate.

This paper focuses on a crucial aspect of risk identification. In recent decades, serious injuries and fatalities (SIFs) have declined at a much slower rate than less serious workplace incidents^2^. Moreover, reducing the overall incidence frequency does not necessarily reduce the SIF frequency in a proportional way. Analyzing SIF-causing incidents alone may not provide insights necessary for the effective mitigation of the SIF risk, given their limited frequency of occurrence. These considerations warrant that incidents that have the potential to give rise to SIF but did not because of chance be given emphasis during risk identification and assessment. Hence, this study deals with the automatic identification of incidents involving PSIF (Potential Serious Injuries and Fatalities). We devise a machine learning based binary classifier that can predict whether a given incident record describes a PSIF incident. We do so by combining a transformer^3^ model based on BERT^4^ (a deep learning model) with XGBoost^5^ (a tree-based model).

Our key contributions are summarized below.

- This work constitutes the first study on automatic risk identification from incident records. We devise an approach for identifying PSIF (Potential Serious Injuries and Fatalities) incidents by combining deep learning based natural language processing (NLP)^6^ with traditional machine learning^7,8^.
- We leverage various techniques ranging from transformers to term frequency based embeddings to combine the textual, numerical, categorical and other types of fields of safety incident records. In particular, we define a novel textual field type based on the size of the vocabulary and encode fields of this type differently than free-text fields. To the best of our knowledge, no existing work applies our multi-faceted approach to safety incident data.
- The data available for training the classifier contains a very small number of manually labeled incident records. We mitigate this problem by employing weak labeling to improve the label coverage of the original training data.
- In order to prioritize detecting PSIF records over avoiding the misclassification of non-PSIF records, we utilize the $F_{\beta }$ metric^9^ with $\beta > 1$ for hyperparameter tuning. As far as we know, we are the first to explain the rationale of how this metric is a suitable choice for developing a safety incident related solution and the first to use it in this context.
- We complement the development of a machine learning based classifier with Exploratory Data Analysis (EDA) and present interesting insights about (PSIF) incidents derived using it.

The rest of the paper is structured as follows. In “Related work section, prior work on risk identification and assessment using machine learning is reviewed. “Dataset section describes the dataset used for this research including the labeling process and discusses insights gained using Exploratory Data Analysis (EDA). “Proposed approach section is devoted to our approach for the classification task pertaining to risk identification. “Experiments section describes experimental results. Section Discussion section provides a discussion, especially of the results. Section Conclusion section concludes the paper.

## Related work

The prior work on risk assessment (including risk identification) using machine learning can be categorized in multiple ways. In^10^, the literature is classified on the basis of several aspects such as the industry type, the kind of data used for risk assessment, and the machine learning method(s) used. In our summary of the existing machine learning based risk assessment work, we segregate the approaches based on the data type(s) involved while also mentioning the industry type as well as machine learning method(s) employed. Sections Risk assessment from textual data section and Risk assessment from image data section pertain to related work involving textual data and image data respectively; “Risk assessment from categorical and numerical data section discusses work involving categorical data and numerical data.

### Risk assessment from textual data

Robinson et al. use Latent Semantic Analysis (LSA) to efficiently identify safety reports of a similar nature^11^. In^12^, Latent Dirichlet Allocation (LDA), a topic modeling technique, is applied on textual descriptions of rail accidents to predict the severity of rail incidents. Marucci-Wellman et al. employ several traditional machine learning algorithms including Logistic Regression (LR) and Support Vector Machines (SVM) on textual narratives describing how workers’ injuries occurred^13^. In^14^, a bidirectional Recurrent Neural Network (RNN) is used to categorize qualitative feedback collected from the public on disaster risk reduction. In^15^, near-miss events arising in construction sites are classified from the engineering/construction reports describing them using BERT (Bidirectional Encoder Representations from Transformers)^4^, a pre-trained neural model based on the transformer architecture^3^.

### Risk assessment from image data

Alawad et al. deploy a Convolutional Neural Network (CNN) on images of passengers at railway stations images to identify falls^16^. In^17^, a model based on VGG-16^18^, a CNN-based model, is developed to detect safety guardrails at construction sites from image data.

### Risk assessment from categorical and numerical data

In^19^, decision trees are employed for analyzing construction fall accidents on data containing categorical and numerical fields. They extract rules associating attributes such as the fall distance and fatality/injury cause to the degree of injury. In^20^, a deep neural network is used for risk assessment involving an oil and gas drilling rig. The model aims to predict risk increase or decrease as the system conditions described by numeric performance indicators change. Cortez et al. use a form of RNN called LSTM (Long Short-Term Memory)^21^ to predict emergency events caused by incidents such as murder and robbery from police-obtained time series data^22^.

#### Observations

- The incident records that we work with consist of varied fields involving textual descriptions as well as categorical data and numerical information. Hence, our work falls in both the first and third categories above (Sections Risk assessment from textual data section and Risk assessment from categorical and numerical data section respectively).
- We note that none of the existing approaches performs risk identification from incident reports from a PSIF standpoint. The prior work that we find the closest to ours is by Fang et al.^15^. While near-miss events, which are the subject of attention in^15^, do bear some similarities with PSIF, the focus of^15^ is the classification of events that are already flagged as near-miss events. They do not automatically identify near-miss events. We, on the other hand, automatically identify incidents involving PSIF.
- In recent years, transformer-based deep learning models^3^ have emerged as the powerful machine learning tool for a number of tasks including text classification^23^. However, the existing literature on exploiting them for risk identification is very limited. Moreover, this is the first work on utilizing transformers in conjunction with traditional machine learning methods for creating a vector representation of an incident record composed of many heterogeneous fields.

## Dataset

VelocityEHS, the employer organization of the authors, offers a software solution for safety incident management. The customers of VelocityEHS who use this software record details about incidents as part of their safety management practices. This database has the incident information of over 600 customers over many years. Our dataset is composed of the incident record data of a subset of the customers who consented to our ML/AI development use of their data, spanning 11 years (from 2012 to 2022).

**Table 1:** Types of columns with examples.

Column category	Description	Example column name and value
Textual	A column containing unstructured text	Title: “Resin spilled in QC office on floor and work bench. No release of chemicals to the environment.”
Quantity-based	A column containing numeric values and associated textual information such as units	OnsiteAreaAffected: “16 sq ft”
Categorical	A column with a small number of unique textual values	Consequence: “Moderate”
Date and time	A column containing date and time information	DateOfOccurence: “2018-06-29 22:00:00”

Our dataset comprises nearly 2.2 million records (rows), each of which provides information about an incident. The incidents are described via over 50 diverse columns (also referred to as fields). These columns can be divided into textual columns, quantity-based columns, categorical columns, and date and time columns. Table 1 defines and illustrates these column categories.

### Data labeling

The data contains a very small quantity of incident records manually flagged as PSIF records. This labeled data was obtained via a multi-step process. First, we acquired a very small amount of incident records that are tagged as PSIF by our customers who agreed to their incident data being used for this work. Please note that the vast majority of customers do not allocate resources for manually processing incidents for PSIF identification and do not have any positive data to share. Since there was inadequate clarity and visibility into customers’ PSIF tagging process and its reliability, we brought together a panel of three occupational safety experts from diverse backgrounds to collectively review these incident records. The three experts shared their perspectives on each incident and discussed among themselves to confirm whether each incident is correctly labeled as PSIF. Almost all the incident records tagged as PSIF by the customers were unanimously deemed to indeed warrant the PSIF tag by our panel of experts and were labeled in our dataset accordingly. The panel was unsure about two incident records, prompting us to leave them unlabeled.

There were two types of judgements involved in the labeling of the incidents by the expert panel.

- Objective judgement: This type of judgements applies to cases in which the panel could conclude with a high degree of certainty that facility personnel were really fortunate that someone was not hurt in the incident. An incident exemplifying such cases is one involving a projectile that passes by someone, missing them by chance. Another example is an arc flash incident from electrical equipment that was not properly isolated; only due to sheer luck, no one walked by it on the day of the incident. These are incidents that the panel of experts was able to categorize as potential SIF (PSIF) incidents with total confidence. An overwhelming majority of the incidents fell in this category.
- Subjective judgement: This is applicable to cases that require a level of imagination to be judged. An illustrative case is an employee suffering from migraine headache, choosing to work nevertheless, and as a result, making a benign, inconsequential mistake. Here, the expert panel needs to adjudicate whether it would have been possible to make a different kind of a mistake with (potentially) severe consequences and whether the fact that they worked while having a headache constitutes a PSIF incident. Such incidents are considerably more challenging for labeling, and the collective knowledge/experience of the senior experts on the panel was relied upon to label them.

Given that our binary classifier is aimed at identifying PSIF, the incident records manually labeled by the experts as PSIF serve as positive training data. Out of the  2.2 million incident records, the number of PSIF-labeled records is merely 2734, amounting to only 0.12% of the records labeled as positive. Manually labeled negative data, meaning incident records identified by subject matter experts as not PSIF, is unavailable. We note that an incident record not labeled as PSIF is not necessarily a non-PSIF record, as not all available incident records have been assessed by experts for identifying PSIF records; the absence of the PSIF label may be attributable to the record not having been manually assessed as opposed to the record having been evaluated and not found to be a PSIF one.

### Data insights

In addition to developing a machine learning powered classifier, we perform Exploratory Data Analysis (EDA) on our dataset. EDA refers to the process of performing investigations using statistics and graphical representations to discover patterns in the data and gain insights without (or before) doing any predictive modeling. We discuss interesting insights obtained using EDA in this subsection.

The textual column category is associated with more columns than any other category. To provide our readers some insight into textual columns, we perform a word-level frequency analysis. For a textual column, for each word present across its entries in the incident records, we compute the number of entries in which it is present as its frequency. We adopt this method of frequency calculation over simply finding the number of occurrences of each word across entries in order to limit the contribution of an entry to a word’s frequency to 1 irrespective of how many times the word is present in the entry.

Table 2 shows the five most frequent words overall and for PSIF incident records for select textual columns. We chose the columns based on factors such as the missing value percentages and importance to the task at hand. The two most frequent words across all incidents for TaskAtTimeOfIncident are “walking” and “truck”, whereas their counterparts for PSIF incidents are “plant” and “ammonia”. The most frequently encountered InjuryIllnessType word is “strain” for all incidents but “laceration” for PSIF incidents. For AccidentAgent, “equipment” is the most frequent word for all incidents as well as PSIF incidents. However, “chemical”, the second most frequent word for PSIF incidents, is absent from the top five words for all incidents. For the BodyPart column, specifying the body part affected by the incident if any, “hand” and “fingers” are among the top four words for all incidents as well as PSIF incidents.

**Table 2:** Most frequent words in select textual columns.

Column	Most frequent words across all incidents	Most frequent words across PSIF incidents
TaskAtTimeOfIncident	walking, truck, driving, loading, moving	plant, ammonia, truck, loading, walking
AccidentAgent	equipment, tool, pallet, hand, surface	equipment, chemical, object, surface, tool
InjuryIllnessType	strain, laceration, contusion, sprain, bruise	laceration, strain, bruise, burn, sprain
Title	hazard, employee, injury, left, fire	door, floor, leak, hazard, water
BodyPart	right, left, hand, fingers, back	left, right, fingers, hand, ankle

**Figure 1:**
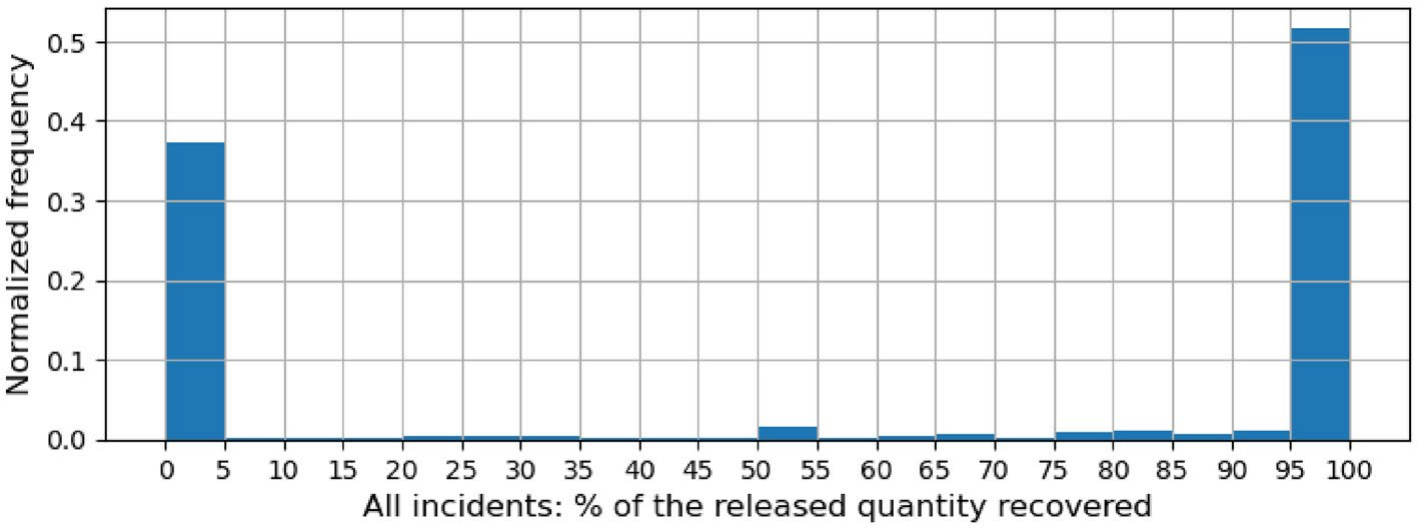
Histogram of the ratio (%) of QuantityRecovered and QuantityReleased across all incidents.

**Figure 2:**
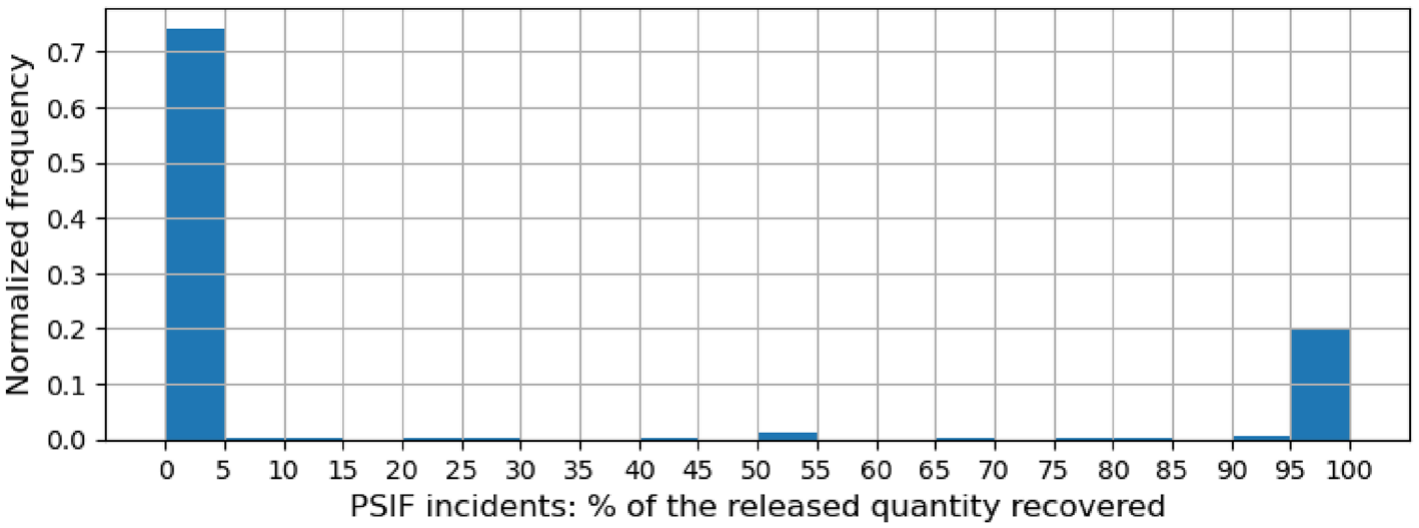
Histogram of the ratio (%) of QuantityRecovered and QuantityReleased across PSIF incidents.

**Figure 3:**
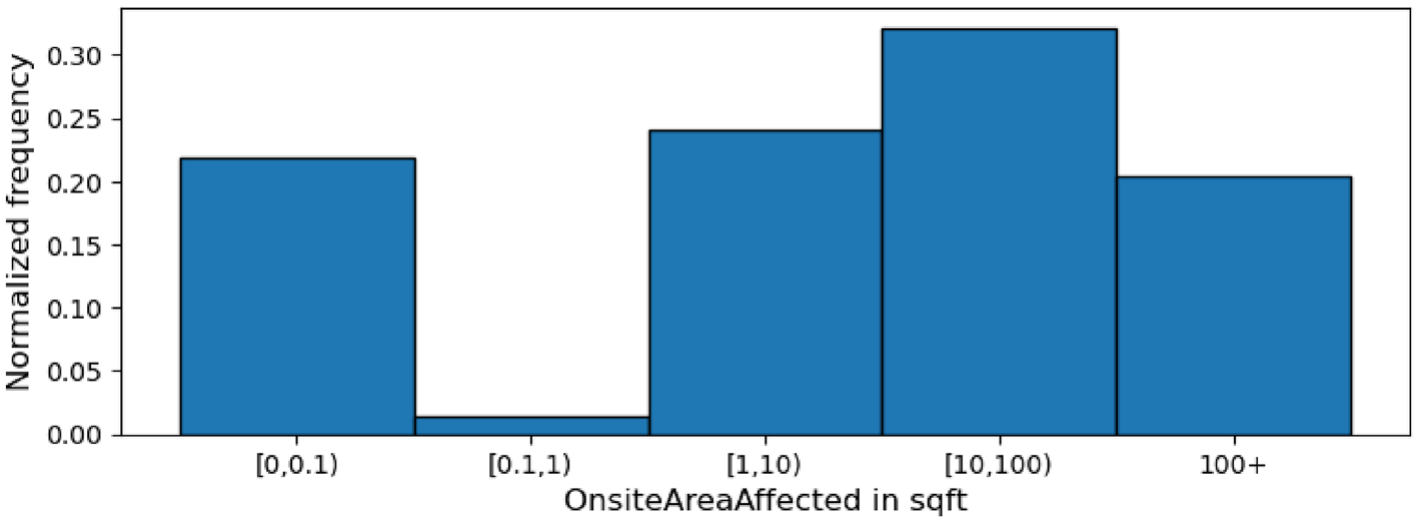
Histogram showing the distribution of OnsiteAreaAffected’s values across all incidents.

**Figure 4:**
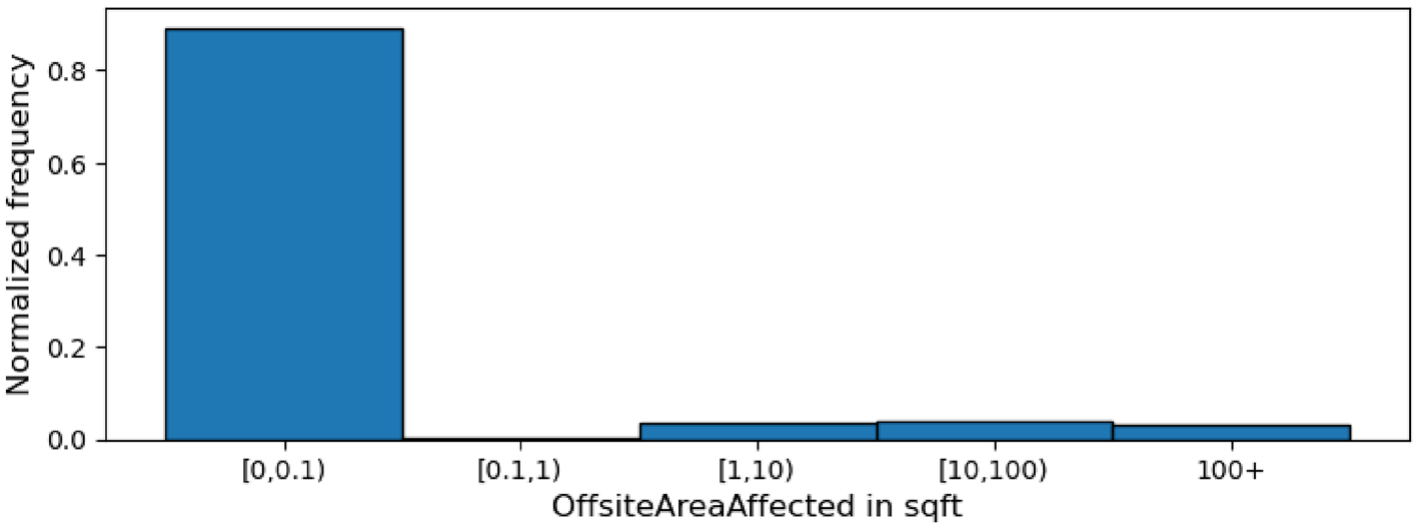
Histogram showing the distribution of OffsiteAreaAffected’s values across all incidents.

QuantityReleased and QuantityRecovered are quantity-based columns that specify the quantities of potentially hazardous or harmful material released on account of the incident and subsequently recovered respectively. We analyze what % of the quantity of materials released gets recovered. Figures 1 and 2 show the distribution of this % for all incidents and PSIF incidents respectively. Across all incidents, in over half the incidents, almost all (95+%) of the quantity of materials released is recovered. However, for PSIF incidents, only in 20% of the incidents, almost all of the release quantity is recovered. On the flip side, almost none (less than 5%) of the released quantity is recovered in 37% of all incidents but over 70% of PSIF incidents. Both of these findings confirm the relative severity of PSIF incidents.

Another pair of related quantity-based columns are OnsiteAreaAffected and OffsiteAreaAffected, providing the sizes of the onsite and offsite areas affected by the incident respectively. Figures 3 and 4 are histograms displaying the distributions of the incident-affected onsite and offsite areas respectively. In line with our expectation, the number of incidents that affect a non-trivial amount (more than 0.1 square feet) of onsite area is considerably higher than the number of incidents affecting a non-trivial amount of offsite area. More specifically, less than 25% of the incidents affect a trivial amount ([0–0.1] square feet) of onsite area. In contrast, over 80% of the incidents affect a trivial amount of offsite area.

**Figure 5:**
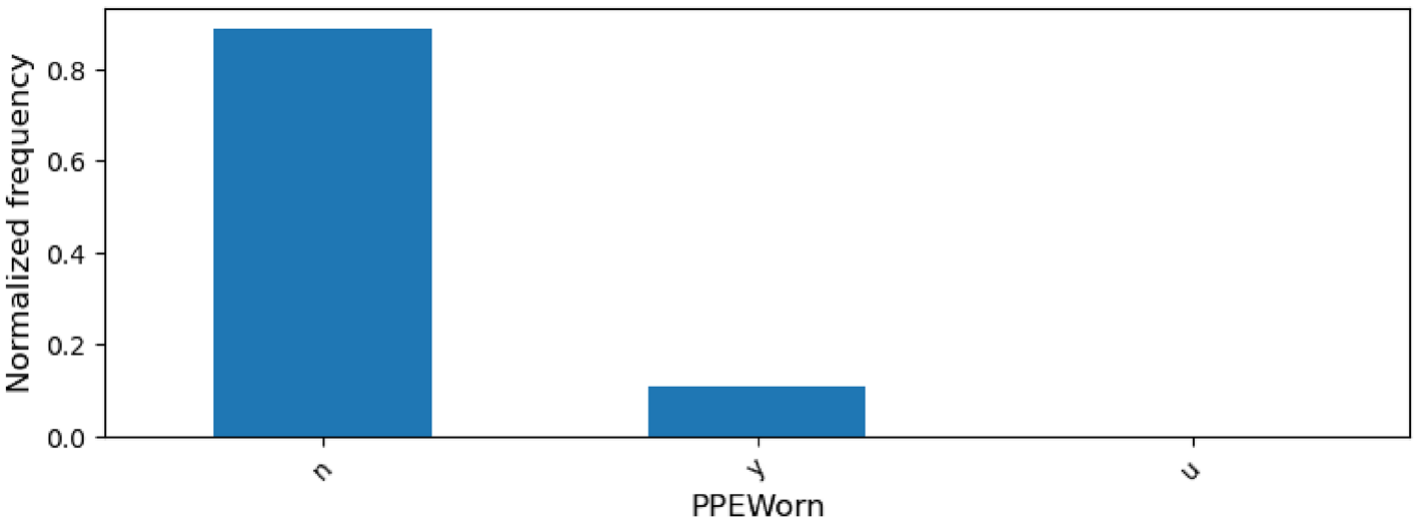
Histogram showing the distribution of the PPEWorn column’s values across all incidents.

We illustrate the categorical column category using PPEWorn, a column specifying whether PPE (Personal Protective Equipment) was worn at the time of the incident. Figure 5 shows the distribution of the PPEWorn column’s values, namely ’n’ (no), ’y’ (yes), and ’u’ (unknown), across all incidents. It can be observed that PPE was not worn in the vast majority (over 80%) of the cases. The distribution of PPEWorn’s values across PSIF incidents is similar.

**Figure 6:**
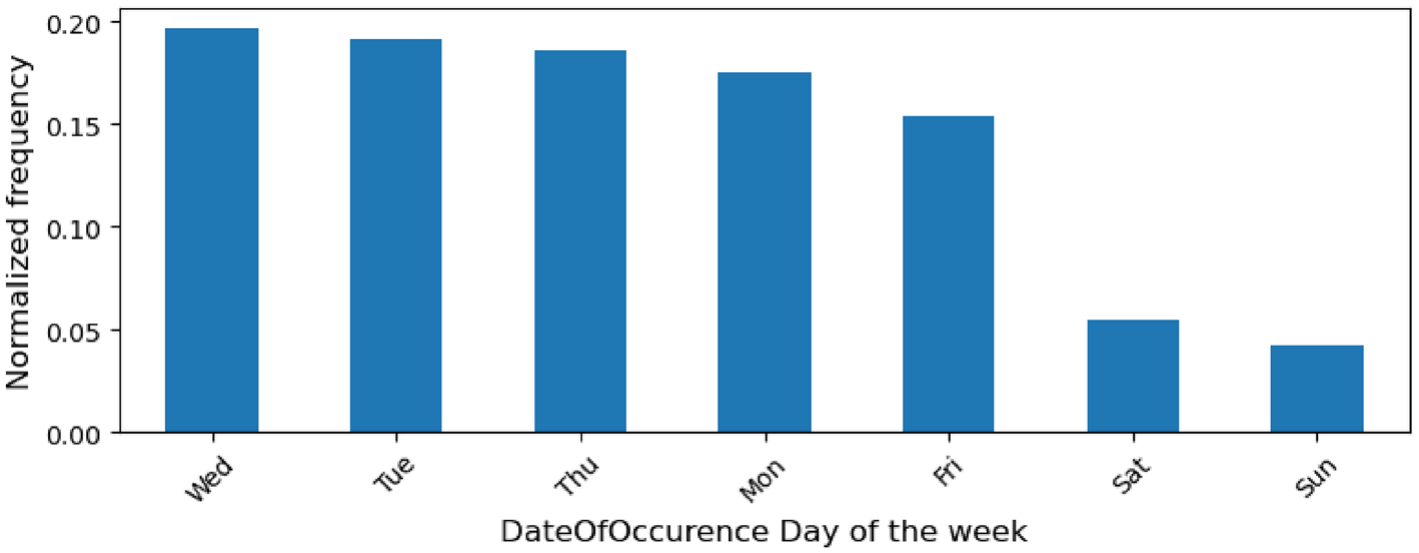
Histogram of the day of the week of DateOfOccurence across all incidents.

One of the date and time columns present in the dataset is DateOfOccurence, which specifies when exactly the incident occurred. Figure 6 is a histogram that provides the distribution of the day of the week of DateOfOccurence across all incidents. Friday is found to be the least incident-prone week day. Among the other week days, none is substantially more incident-prone than another. Friday remains the least frequent week day for PSIF incidents too.

**Figure 7:**
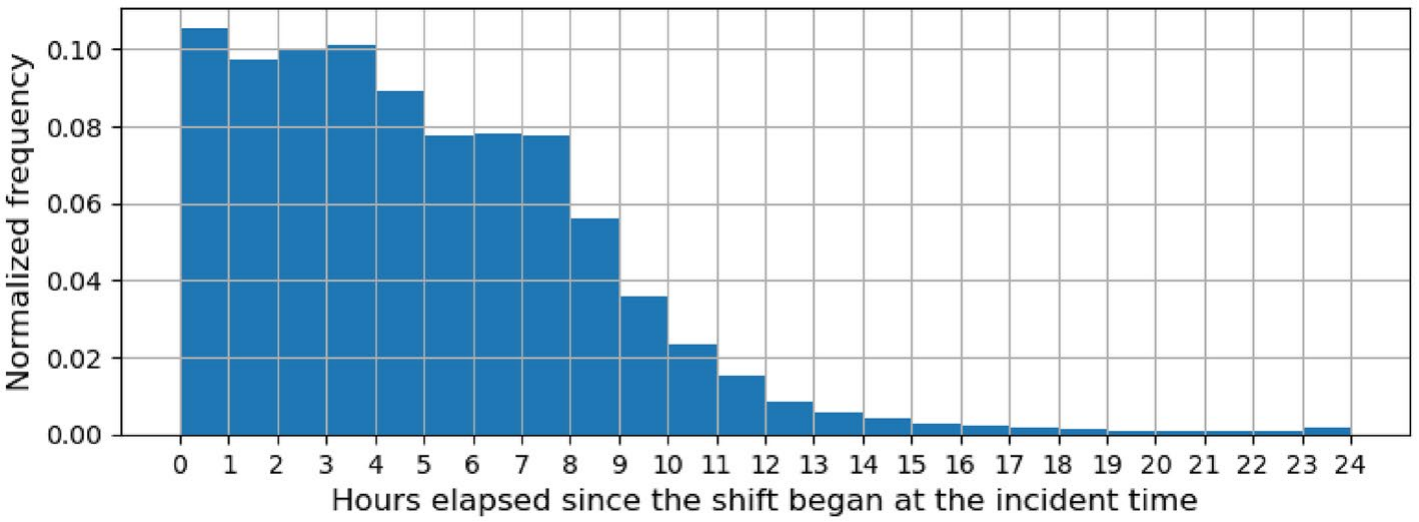
Histogram showing the distribution of the hours elapsed between ShiftBegan and DateOfOccurence across all incidents.

Figure 7 shows the distribution of the differences between DateOfOccurence and ShiftBegan (when the shift associated with the incident time began) in hours across all incidents. The number of incidents is seen to decrease as the time gap increases. An incident is the most likely to take place in the first hour of a shift. With regard to PSIF incidents, more than half of them occur within the first 6 h of a shift. The same also holds true for all incidents.

### Data quality

**Figure 8:**
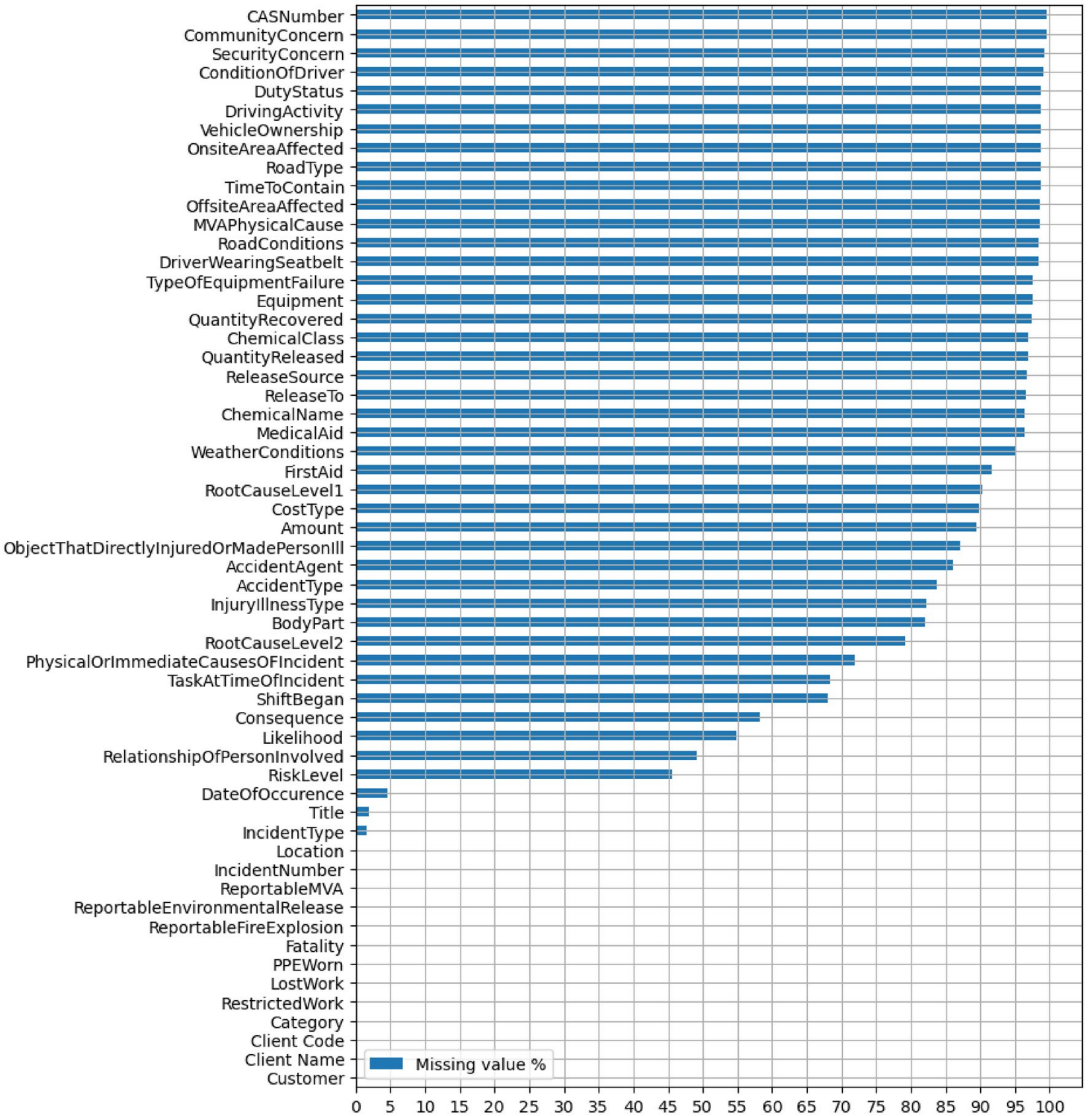
The columns/fields in the dataset and the associated missing value percentages.

There are two factors that adversely affect the quality of the data. The most consequential issue is the extent of missing values. Figure 8 shows the percentage of missing values for each column. As seen in the figure, 26 (46%) columns have over 90% missing values each, and 33 (58%) columns have over 80% missing values each.

The other data quality related challenge pertains to unhelpful and noisy information. Some of the data is not very informative or actionable, despite being outwardly relevant. For instance, an overwhelming majority of the values in the columns ReportableEnvironmentalRelease, reportableFireExplosion, and ReportableMVA are u, meaning unknown. The data also contains some erroneous information. This is illustrated by the fact that some records (rows) have a higher value for QuantityRecovered than for QuantityReleased, even though as it is clearly impossible to recover more quantity of materials than the amount released.

## Proposed approach

**Figure 9:**
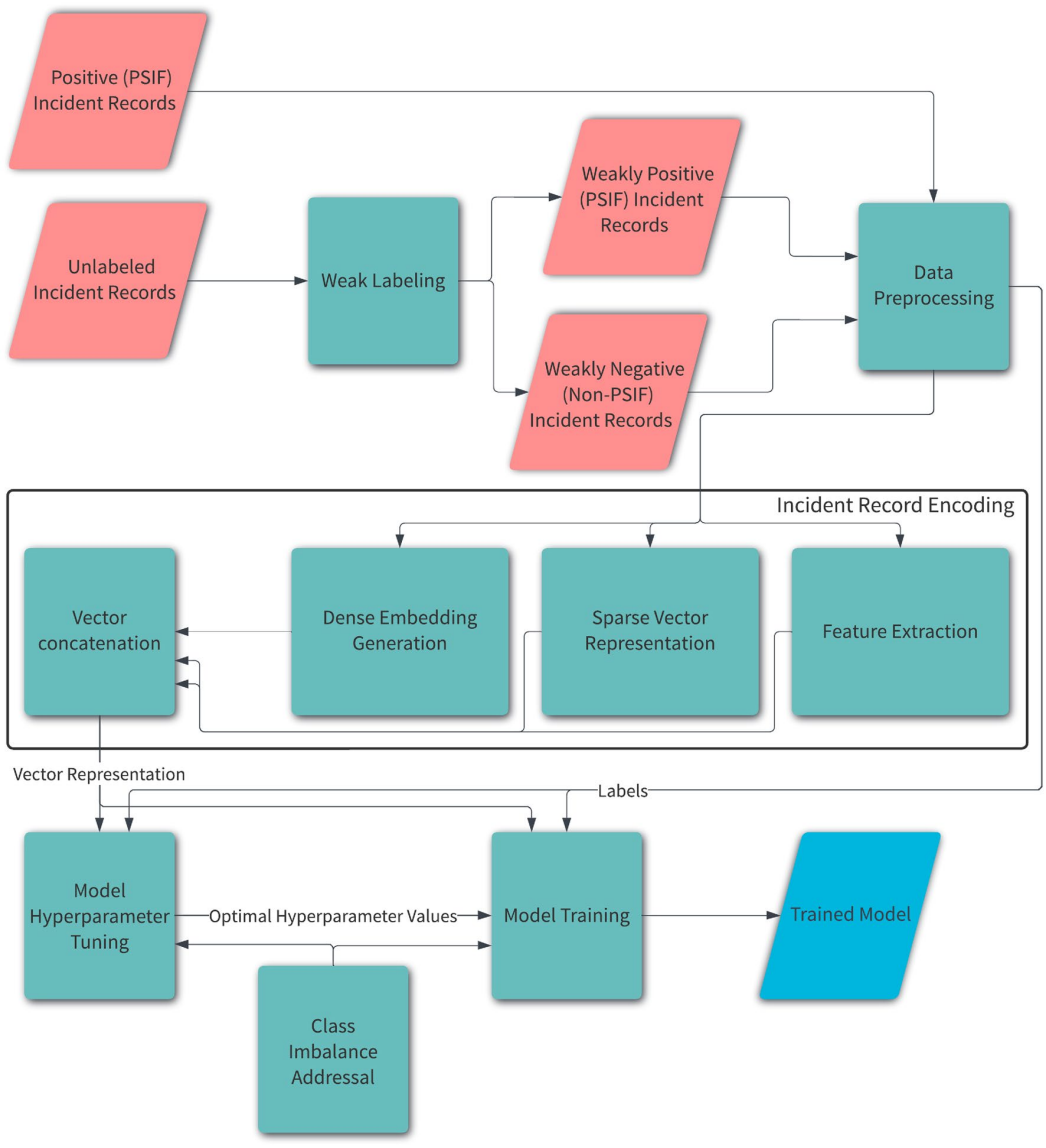
Training the proposed PSIF classifier.

We combine natural language processing^6^, deep learning^23,24^, and traditional machine learning^7,8^ to develop a classifier that predicts whether a given incident record is PSIF or not. Figure 9 shows the steps involved in training a machine learning model for the lessons-learned incident classification. Descriptions of the major steps are provided below. We end this section by discussing the inference process.

### Weak labeling

Weak supervision through the creation of weak labels is a useful strategy for mitigating labeled data scarcity^25^. We utilize weak labeling to alleviate two issues pertaining to labeled data, namely the absence of negative data and the extremely small volume of positive data. With the help of our subject matter experts, we identify categorical columns that are each highly indicative of PSIF incidents. We label all incident records for which any of these columns has an affirmative value corresponding to the incident occurrence as positive (PSIF), thereby augmenting the positive data from  2700 to  70000. All records other than these weakly labeled positive records and the manually labeled positive records are treated as negative (non-PSIF).

### Data pre-processing

We pre-process the relabeled data by filtering out rows and columns before carrying out classification. The first pre-processing step that we perform is to filter out rows based on the temporal distribution of the incidents. Temporal distribution serves as a proxy metric for expected data quality as recent data is usually more reliable. Specifically, we drop records based on the year of incident occurrence. Owing to the fact that positive data is in really short supply, we use different filtering thresholds for positive and negative records such that positive records are dropped very conservatively. Moreover, we do not apply the same filtering to all negative records. We take into account a set of columns deemed important by our subject matter experts. We split the negative data into two (non-overlapping) groups such that one group contains the records wherein the values for all important columns are missing. We apply a considerably more liberal (higher) year of incident occurrence threshold for filtering out this group, whereas we drop records conservatively from the other group wherein every record has a non-missing value for at least one important column.

In the second pre-processing step, we identify columns that are irrelevant for our classification and clustering tasks based on conceptual analysis and domain understanding. Examples of the columns thus dropped are ’Client Name’, ’IncidentNumber’, and ’Client Code’. Additionally, we filter out columns on the basis of low coverage, which is equivalent to high missing values. Specifically, a column is removed if it is less than 20% populated overall (meaning a missing value % of over 80) and less than 5% populated among the positive records. Columns flagged as important by our team of subject matter experts are whitelisted (i.e. retained regardless of the overall coverage and positive data coverage). All columns used for weak labeling are removed to prevent the classifier from predominantly learning the rules used to weakly label the data.

The data resulting from the application of the row filtering and column filtering has  783000 rows and 28 columns.

### Incident record encoding

Encoding an incident record into a vector is a crucial component of our approach to classifying whether it is PSIF. We identify and conceptualize two subcategories of textual columns to that end, giving rise to a total of five diverse categories. We employ a different encoding scheme for the columns of each of these categories. In addition to utilizing the established free-text category, we devise a novel textual category termed small-vocab that relates to the size of the vocabulary, i.e. the number of unique words across rows in a column. Using a different encoding method for the columns of this new category leads to more compact vector representations than considering them free-text or categorical columns and encoding them accordingly. Compact representations are desirable for the effective and efficient training of the downstream machine learning model used for classification owing to the curse of dimensionality^26^. Table 3 defines and illustrates the two subcategories of textual columns.

**Table 3:** The subcategories of textual columns with examples.

Column category	Description	Example column name and value
Free-text	A column containing unstructured text with a reasonably large vocabulary	Title: “Resin spilled in QC office on floor and work bench. No release of chemicals to the environment.”
Small-vocab	A textual column with a small vocabulary size but too many unique values for a categorical column	TypeOfEquipmentFailure: “Mechanical Failure - Gas Coolers”

We encode the 28 column entries (belonging to the 5 column categories) that make up an incident record through three different methods detailed below. The resultant vector representations of these column entries are concatenated to produce a single vector representing the incident record.

#### Dense embedding generation

We utilize advanced natural language processing (NLP) techniques^6^ based on deep neural networks to encode free-text columns, which contain unstructured text with a reasonably large vocabulary. More specifically, we employ a transformer^3^ based model aimed at language understanding to generate a dense, contextual embedding of a free-text column entry. The transformer model that we opt for is DistilBERT^27^, a lighter version of BERT, since A) it is faster than BERT and B) has a smaller hidden state size, resulting in a more compact vector representation. We use the final-layer hidden state of the CLS token from the DistilBERT tokenization as the text embedding. It outperforms mean-pooling the hidden states at the final or penultimate layer.

We also explore another method of encoding free-text columns that leads to a considerably smaller vector representation and hence a more efficient classifier. This method involves the use of GloVe^28^, a distributional word embedding technique. We average the pre-trained GloVe embeddings of the words of a free-text column entry to compute its vector representation. The words not present in the GloVe vocabulary are disregarded. The fact that GloVe word embeddings are more than twice as small as the DistilBERT final-layer hidden states lends efficiency to a classifier trained using this encoding scheme for free-text columns compared to the primary, transformer-based method described earlier.

#### Sparse vector representation

We apply one-hot encoding^29^ to categorical columns in view of the fact they (each) have a small unique value count. First, in each categorical column, each entry with a frequency of less than 5 is replaced with ‘other’ so as to prevent a potentially long tail of infrequent entries from inflating the size of the vocabulary and consequently the vector representations. We then carry out one-hot encoding that produces a vector for each entry such that it has the value 1 at the index of the entry in a fixed, ordered list of the unique column entries. We store the ordered list and the list of entries that are lumped into the ’other’ bin per column so that we can use them to encode the categorical fields of an unseen incident record at the time of inference.

Given that the size of a small-vocab column’s vocabulary is small, we use term (word) frequency based vector representations for the columns of this category. For each column, we ignore terms that are each present in less than 5 entries. A small-vocab column has too many unique values for one-hot embedding to produce adequately small vector representations. Treating it as a free-text column and thus encoding it by generating dense embeddings would also result in larger vectors than the chosen method does. We store the ordered vocabulary list and use it to encode unseen records during inference.

#### Feature extraction

For quantity-based columns, which typically contains area, volume, or time information, we first employ regular expressions to separate the numerical part (associated with a quantity) of each entry from the textual part (containing the unit information). We then remove non alphanumeric characters from the textual parts of the entries and apply the term frequency based encoding, which amounts to one-hot encoding in this case, on it. Terms that are each present in the textual parts of less than 5 entries are ignored. The vectors resulting from this encoding are concatenated with the numbers extracted using regular expressions to produce the composite vector representations for a quantity-based column.

For the date and time columns, we use various properties such as the day of the week, day, month, and hour.

### Model hyperparameter tuning

Considering that tree-based models often outperform deep learning on tabular data^30^ and that XGBoost^5^ achieves state-of-the-art results on many tabular datasets^31^, we choose XGBoost as the classification model. XGBoost entails a number of hyperparameters including the maximum depth of a tree and the L2 regularization term on weights. We employ an automatic method called Tree-structured Parzen Estimator (TPE)^32^ to find the best estimates for some of them (and two hyperparameters not linked with XGBoost discussed in Sections Model training section and Inference section). This method entails minimizing an objective function parameterized by the hyperparameters sought to be estimated. Our objective function entails first performing model training (discussed in “Model training section) using the input hyperparameter values and then evaluating the resultant trained model. The metric that we use to evaluate the trained model is $F_{\beta }$^9^ , where $\beta > 1$.

1 $$\begin{aligned} F_{\beta } = \frac{(1 + \beta ^2) \cdot (\text {precision} \cdot \text {recall})}{(\beta ^2 \cdot \text {precision}) + \text {recall}} \end{aligned}$$

The rationale behind this novel choice is that, after consultation with safety subject matter experts, we learned that it is more important to not miss PSIF incidents than to not misclassify a non-SIF incident as SIF. This translates to the maximization of recall being more desirable than the maximization of precision. Popularly used metrics like $F_1$ and accuracy treat precision and recall (or false positives and false negatives) equally. $F_{\beta }$, however, allows us to weigh recall higher (or lower) than precision through the choice of the $\beta$ value. We choose a $\beta$ value greater than 1 to give a higher precedence to recall than precision. We empirically find $F_2$ to the best objective function for automatic hyperparameter estimation using TPE. The objective function that we minimize through TPE to find the best hyperparameter values is $-F_{\beta }$.

### Model training

Using the hyperparameter values obtained through the previous step, we create a classifier and train it using the vector representations of the incident records and the corresponding binary labels (denoting whether a record is PSIF or not). We make use of early stopping for this training (as well as for the repeated training carried out for hyperparameter tuning), as it can help prevent overfitting and save training time.

Despite weak labeling expanding the positive data and post-processing disproportionately filtering out negative data, out of the  783000 incident records selected as per “Data pre-processing section, only  47000 are positive. This implies extreme class imbalance with the positive records to negative records ratio of  6:100. We mitigate the impact of this class imbalance by increasing the weight of the positive samples in the model during training. The weight by which the positive records are scaled (up) is a hyperparameter automatically estimated as described in “Model hyperparameter tuning section.

### Inference

**Figure 10:**
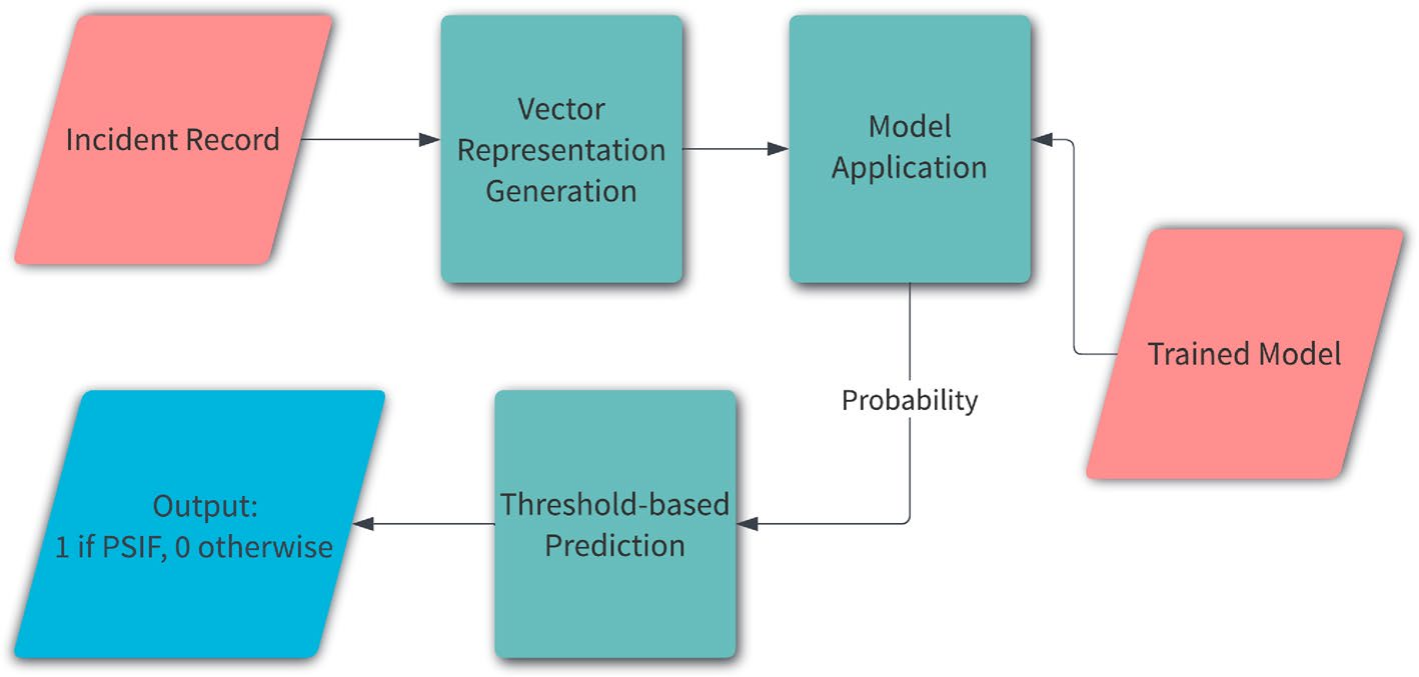
Making predictions using our PSIF classifier.

Figure 10 shows the process of inference i.e. prediction for a new (unseen) incident record. We generate a vector representation of the record using the steps detailed in “Incident record encoding section and feed it to the trained XGBoost model. We apply a threshold to the probabilities generated by the model to predict if the record is lessons-learned or not. The threshold is a part of the hyperparameters tuned (as discussed in “Model hyperparameter tuning section).

## Experiments

We evaluate the proposed framework against several baselines. We reserve 20% of the  783000 incident records for testing such that the training set contains approximately the same percentage of samples of each of the two classes as the complete set. While carrying out hyperparameter tuning, we set aside 20% of the training data as the validation set and use the remainder for training. We utilize all of the training data (including the validation set used for hyperparameter tuning) for the final training.

### Baselines

We provide a comparison of the proposed methods against several baseline methods.

#### TFIDF + logistic regression

This baseline is similar to a method used by Marucci-Wellman et al.^13^ in terms of the use of TFIDF (Term Frequency - Inverse Document Frequency) to encode free-text fields^33^ and the use of logistic regression as the binary classification model. The fields of all other types are encoded as described in “Incident record encoding section.

#### TFIDF + XGBoost

In this baseline, all fields are encoded as per “TFIDF + logistic regression section with TFIDF used to encode free-text fields. However, instead of logistic regression, XGBoost is applied on the composite vector representation of incident record for the binary PSIF classification.

#### DistilBERT + logistic regression

In this baseline, DistilBERT is used to encode free-text fields as detailed in “Incident record encoding section. All other fields are also encoded as per “Incident record encoding section. The resultant composite vector representation is fed to logistic regression for the binary classification.

### Evaluation metrics

We report the results with three popularly used binary classification metrics, namely precision, recall, and ${F_1}$. Given the asymmetry between precision and recall in terms of the precedence given, we also use ${F_2}$ to evaluate the classification performance. As seen in Eq. (1), recall carries more weight in ${F_2}$ than in ${F_1}$.

### Results

Table 4 shows the results for the proposed approach and the baselines. We also provide results for two variants of the proposed approach. The variant mentioned in Row 4 of Table 4 is the GloVe-based one described in “Dense embedding generation. In the variant mentioned in row 5, we replace DistilBERT with BERT as the tranformer model.

**Table 4:** PSIF Classification Results.

Approach	$\text{Precision}$	$\text{Recall}$	F_1_	F_2_
TFIDF + Logistic Regression	0.37	0.92	0.53	0.71
TFIDF + XGBoost	0.40	0.96	0.56	0.75
DistilBERT + logistic regression	0.43	0.91	0.58	0.74
Proposed GloVe-based approach	0.46	0.94	0.62	0.78
Proposed BERT-based approach	0.49	0.93	0.64	0.79
Proposed DistilBERT-based approach	0.48	0.94	0.63	0.79

The DistilBERT-based encoding of free-text columns outperforms TFIDF, as shown by the two F scores in rows 1 and 3 of Table 4. Unlike TFIDF, DistilBERT, which is a transformer based model, can help generate contextual, semantic vector representations that take into account the word order.

XGBoost yields better results than logistic regression as the binary classification model (rows 1 and 2 of Table 4). This is likely due to a significant number of categorical fields in the incident data. XGBoost is also found to be more computationally efficient in terms of the training time.

The proposed methods utilizing transformers for encoding free-text fields and XGBoost as the binary classification model produce the best results overall. The more efficient proposed approach involving the averaging of fixed word embeddings given by GloVe^28^ slightly underperforms them. Using BERT as the transformer model in the proposed approach produces almost identical results to using DistilBERT. However, the former incurs a greater computational cost on account of BERT giving rise to a longer vector representation.

## Discussion

Since ours is the first work on automatic PSIF identification from incident records, there are no existing methods to compare against. The baseline methods described in “Baselines section are methods that we have designed, with a view to showing how the chosen approach compares against alternatives in terms of vector representation generation and the subsequent classification.

While we prefer maximizing recall over precision, precision still holds considerable value. Hence, for each method, along with precision and recall, we also report ${F_1}$ and ${F_2}$, each of which is a function of both precision and recall. The most apt metric for our scenario is ${F_2}$, because it weighs recall more than precision. Table 4 shows that the ${F_2}$ value for the proposed approach is 4 percentage points higher than the best baseline method (TFIDF + XGBoost).

Also, there is a trade-off between precision and recall that can be controlled by simply varying the threshold applied on the probability generated by a trained model to make the prediction. The lower the threshold, the higher the recall and the lower the precision. Hence, we can trivially improve our recall past that of the TFIDF + XGBoost baseline by simply choosing a lower threshold.

Having discussed how we can control the trade-off between precision and recall, we now delve into which of the two should be traded for the other. PSIF identification is an asymmetrical problem in the sense that the cost of not identifying a PSIF incident (a false negative) is not the same as labeling a non-PSIF incident PSIF (a false positive). The reason is that every PSIF incident that reoccurs in the future has a chance to materialize as SIF and thereby result in severe harm. If the previous occurrence of a PSIF incident is successfully identified by a solution like ours, future SIFs can be prevented by the control measures that are implemented following PSIF identification. However, missing to identify a PSIF means that it is likely going to go under the radar, and no control measures will prevent its recurrence, leaving the risk of future harm uncontrolled.

On the other hand, given that each nomination of PSIF by our software solution is reviewed by the customers, the cost of false positives (or low precision) is added work to review nominated incidents. This cost is insignificant for the following reason. For a system such as ours with close to 50% precision, for every two incidents flagged by our system, a manual review by the customer will uphold one as PSIF and disregard the other. Given that PSIF incidents are rare occurrences, the % of the total number of incidents flagged by our system for manual evaluation is small, since it is twice the (small) actual PSIF incident %. From a pure efficiency point of view, the proposed system enables our customers to reduce the volume of data that needs to be manually reviewed for finding PSIF incidents from 100% of the incident records down to a significantly smaller number of incidents flagged by the system.

## Conclusion

This paper explores machine learning powered automatic identification of potential serious injuries and fatalities (PSIF) from incident reports to assist in risk identification with the purpose of improving workplace safety. We propose a novel approach for this PSIF classification that involves encoding heterogeneous fields that make up an incident record through several different methods and applying XGBoost on the resultant vector representation to perform binary classification. The encoding methods that we exploit range from the generation of dense embeddings through a transformer model based on BERT from free-text fields to combining regular expressions with term frequency based encoding for quantity-based columns. We factor in our safety subject matter experts’ guidance of prioritizing the maximization of recall over precision through the use of the F2 metric for hyperparameter tuning. Our experiments find the proposed methods to be superior to multiple baselines.

## Data Availability

VelocityEHS provides software solutions in Environmental, Health, and Safety space. One of our modules is safety incident management, and it has been used by numerous customers to report their safety incidents, track them, and manager their corrective control actions. In the process to develop our models, evaluation, and testing, we have used the data from our customers with their consent. The privacy, security, and consent of our customers is of utmost importance. Hence, we are unable to share the entire dataset publicly. However, small samples of our data can be requested by emailing Pulkit Parikh, as we have prior consent from select customers for this purpose. All the data used in this study was anonymized by removing the fields about personal information and the anonymity of the remaining data was further verified using best practices. We were granted the administrative permission to use the raw data in this study based on the guidelines of the compliance officer at VelocityEHS.
